# Prediction of Skin Sensitization with a Particle Swarm Optimized Support Vector Machine

**DOI:** 10.3390/ijms10073237

**Published:** 2009-07-17

**Authors:** Hua Yuan, Jianping Huang, Chenzhong Cao

**Affiliations:** 1 Key Laboratory of Theoretical Chemistry and Molecular Simulation of Ministry of Education, Hunan University of Science and Technology, Xiangtan 411201, China; E-Mails: yh_cathy@163.com (H.Y.); czcao@hnust.edu.cn (C.C.); 2 Hunan Provincial University Key Laboratory of QSAR/QSPR, Xiangtan 411201, China; 3 School of Chemistry and Chemical Engineering, Hunan University of Science and Technology, Xiangtan 411201, China; 4 Pharmaceutical Informatics Institute, College of Pharmaceutical Sciences, Zhejiang University, Hangzhou 310027, China

**Keywords:** skin sensitization, guinea pig maximization test, murine local lymph node assay, support vector machine, particle swarm optimization

## Abstract

Skin sensitization is the most commonly reported occupational illness, causing much suffering to a wide range of people. Identification and labeling of environmental allergens is urgently required to protect people from skin sensitization. The guinea pig maximization test (GPMT) and murine local lymph node assay (LLNA) are the two most important *in vivo* models for identification of skin sensitizers. In order to reduce the number of animal tests, quantitative structure-activity relationships (QSARs) are strongly encouraged in the assessment of skin sensitization of chemicals. This paper has investigated the skin sensitization potential of 162 compounds with LLNA results and 92 compounds with GPMT results using a support vector machine. A particle swarm optimization algorithm was implemented for feature selection from a large number of molecular descriptors calculated by Dragon. For the LLNA data set, the classification accuracies are 95.37% and 88.89% for the training and the test sets, respectively. For the GPMT data set, the classification accuracies are 91.80% and 90.32% for the training and the test sets, respectively. The classification performances were greatly improved compared to those reported in the literature, indicating that the support vector machine optimized by particle swarm in this paper is competent for the identification of skin sensitizers.

## 1. Introduction

With the fast development of industry, agriculture and medication, human are exposed to more and more exogenous chemicals, some of which may result in allergic contact dermatitis after accidental or deliberate skin contact. The medical condition of allergic contact dermatitis is known as skin sensitization, which is associated with an alteration of the immune system. According to statistics from the U.S. Bureau of Labor, occupational contact dermatitis is the most commonly reported non-trauma related category of occupational illnesses in the United States [1]. The total annual losses due to occupational skin diseases were estimated to amount to over one billion dollars [2]. Therefore, identification and labelling of environmental allergens is an urgent request from consumer organizations, industry, and governmental agencies to protect people from skin sensitization. In the new European Union (EU) chemical policy REACH, information on skin sensitization potential will have to be provided for any chemicals manufactured or imported in amounts above 1 tonne/year [3].

The guinea pig maximization test (GPMT) and murine local lymph node assay (LLNA) are the two most commonly used *in vivo* models for identification of skin sensitizers. GPMT combines the use of intradermal administration of the compound with and without Freund’s complete adjuvant (FCA), and occluded topical application of the compound a week later [4]. The result of GPMT relies on subjective evaluation of a group of animals and is usually expressed as the dichotomous (sensitizer/nonsensitizer) form. The murine local lymph node assay defines skin sensitization hazard as a function of the ability of the test chemical to provoke lymphocyte proliferation on lymph nodes draining the site of topical application. A substance is classified as a sensitizer if it induces a threefold stimulation index (EC3) or greater at one or more test concentrations. The value of EC3 is continuous and indicates the relative skin sensitizing potency of chemicals, but the majority of published LLNA data nowadays are also in the dichotomous form [5]. Due to the huge number of chemicals with unknown skin sensitization potential, exhaustive animal testing of such chemicals is costly and raises ethical concerns. Therefore, the use of other alternative methods such as quantitative structure-activity relationships (QSARs) is strongly encouraged in order to reduce the number of animal tests. Several legislations have recently emerged to further develop and increase the acceptance of QSARs in assessment of skin sensitization of chemicals [6] and much work has been reported [7–10].

This paper aimed to build a classifier to distinguish skin sensitizers from non-sensitizers based on various compounds with LLNA and GPMT results. When compared to other classification techniques such as discriminant analysis [11], random forest methods [9] and artificial neural networks [12] the support vector machine (SVM) has been proven advantageous in handling classification tasks in cases of high dimensionality of data points. However, the input features (molecular descriptors here) of SVM play a very important role in the classification performance. Not all the molecular descriptors are equally important for a specific classification. Many of them may be redundant or irrelevant. If SVM is implemented without feature selection, the dimension of the input space is very large and non-clean, which will impair the performance of the SVM. The particle swarm optimization (PSO) algorithm is a swarm intelligence method for optimization problems and has been widely applied to feature selection. Compared with other descriptor selection approaches, such as the genetic algorithm (GA) and recursive feature elimination (RFE), PSO is not only much simpler in concept and more computationally efficient [13], but it also exhibits advantages in solving many kinds of optimization problems featuring nonlinearity and nondifferentiability, multiple optima, and high dimensionality [14,15]. Lin *et al.* [16] conducted a thorough study on the performance of PSO as a parameter determination and feature selection technique for SVM. The results based on about ten different data sets adequately confirmed that the performance of SVM+PSO outperforms that of SVM+GA and SVM without feature selection. Therefore, this paper investigates the potential of the support vector machine in combination of the particle swarm optimization algorithm for feature selections in addressing the problem of identification of sensitizers.

## 2. Materials and Methods

### 2.1. Data Set

The Interagency Coordinating Committee on the Validation of Alternative Methods (ICCVAM) issued the LLNA results of 209 compounds, and for some of which, the GPMT results were also available. All the experimental data were obtained within the “spirit” of Good Laboratory Practice guidelines. Fedorowicz [5] cleared out the inorganic salts, natural products and polymers from the ICCVAM list and developed a data set of 178 organic compounds, although it still contains 16 sodium salts which cannot be processed by the Dragon software used to calculate molecular descriptors in this work. Thus, a total 162 compounds were used in this paper after sodium salts were excluded. These compounds pertain to a number of chemical classes, including alkanes, aromatic hydrocarbons, alcohols, amines, acids, esters and so on. All 162 compounds have LLNA results, which indicate 119 sensitizers and 43 non-sensitizers. Furthermore, 92 of 162 compounds also have GPMT data, indicating 71 sensitizers and 21 non-sensitizers. For convenience of expression, the above two data sets with LLNA and GPMT results were denoted as LLNA data set and GPMT data set, respectively. For each data set, two thirds of compounds were randomly assigned as the training set, and the leftovers composed the test set. The information of each data set is shown in Table 1.

### 2.2. Calculation of Molecular Descriptors

Molecular descriptors characterizing molecular structure were calculated in Dragon 5.4 [17]. Twenty blocks of molecular descriptors were embodied in Dragon package. In this paper, only 926 descriptors contained in blocks 1–10, 17–18 and 20 were calculated, with no consideration of 3D descriptors. These descriptors consisted of constitutional descriptors, topological descriptors, walk and path counts, connectivity indices, information indices, 2D autocorrelations, edge adjacency indices, BCUT descriptors, topological charge indices, eigenvalue-based indices, functional group counts, atom-centered fragments, and molecular properties.

### 2.3. Preprocessing of Molecular Descriptors

In order to delete the noisy, irrelevant and redundant information, the calculated 926 molecular descriptors were preprocessed by eliminating: 1) those having same values for greater than 90% of the compounds; 2) those having high correlation coefficients (>0.85) with other descriptors.

Since these molecular descriptors characterize the structural information from extensive perspectives, their magnitudes are quite various. In order to prevent the descriptors in greater numeric ranges from outweighing those in smaller numeric ranges, the original descriptors were scaled to the range [0, 1] using min-max normalization method [18] prior to the next feature selection step with particle swarm optimization (PSO) algorithm. Min-max normalization was realized according to Equation (1):

(1) $${\text{V}}^{\prime }=\frac{\text{V}-\text{min}}{\text{max}-\text{min}}$$

where *min* and *max* are the minimum and maximum values of a descriptor, *V* and *V’* represent the descriptor before and after scaling, respectively.

### 2.4. Particle Swarm Optimization (PSO) Algorithm

Particle swarm optimization is a population-based meta-heuristic algorithm that simulates social behavior such as bird flocking and fish schooling. Since introduced originally by Kennedy and Eberhart [19] in 1995, PSO has been continuously developed and widely applied to solving optimization problems due to its reduced memory requirements and fast convergence [20,21]. Like evolutionary algorithms, PSO performs searches using a population (swarm) of individuals (particles) that are updated from iteration to iteration to find an optimal solution. Each particle, representing a potential solution, is treated as a point in a D-dimension space and its status is characterized by its position and velocity. The position vector (***x_i_***) and velocity vector (***v_i_***) for particle *i* in a D-dimension space can be represented as ***x_i_*** ={***x***_***i***1_, ***x***_***i***2_,..., ***x_iD_***} and ***v_i_*** ={***v***_***i***1_***, v***_***i***2_***,..., v_iD_***}, respectively. Each particle keeps track of its personal best position ***p_i_*** ={***p***_***i***1_, ***p***_***i***2_,..., ***p_iD_***} it has achieved so far and the global best position ***p***_g_ ={***p***_g1_, ***p***_g2_,..., ***p***_g***D***_} that has been found by other neighboured particles in the swarm. At each iteration, ***p_i_*** and ***p*_g_** vector are combined to update the velocity of particle *i* along each dimension, and the velocity is then used to adjust the new position for that particle as given in Equations (2) and (3).

(2) $${\text{v}}_{\text{id}}(\text{new})=\text{w}{\text{v}}_{\text{id}}(\text{old})+{\text{c}}_{1}{\text{r}}_{1}({\text{p}}_{\text{id}}-{\text{x}}_{\text{id}})+{\text{c}}_{2}{\text{r}}_{2}({\text{p}}_{\text{gd}}-{\text{x}}_{\text{id}})$$

(3) $${\text{x}}_{\text{id}}(\text{new})={\text{x}}_{\text{id}}(\text{old})+{\text{v}}_{\text{id}}(\text{new}),d=1,\;2,\;...,\;D$$

where ***w*** is an inertia weight which contributes to balance the global search and local search; ***c*_1_** and ***c*_2_** are two positive constants indicating the cognition learning factor and the social learning factor, respectively; ***r*_1_** and ***r*_2_** are random numbers uniformly distributed in the range [0, 1]. The iteration is terminated if the minimum error criterion (fitness) is attained or the number of iterations reaches the predetermined limit.

Although the basic PSO algorithm presented above was originally designed for continuous problems, attempts have been made to extend it to discrete optimization issues, where the particle position is composed of a set of bits that contain either ‘1’ or ‘0’, indicating being selected or not [22]. Most modified algorithms lose their consistent form or way of evolution exhibited in the continuous particle swarm algorithm. This paper designed the discrete PSO simulating the continuous PSO, where the position and velocity of a particle were updated in continuous space. Only when the new candidate position is passed to the fitness function, it is transformed from the continuous space to the discrete space. Supposing that the particle position values is limited to interval [0, 1], conversion can be accomplished by mapping the values hitting in the interval [0, 0.5) to 1, and other values to 0.

In general, the number of molecular descriptors selected for QSAR modeling is considered one of the important factors responsible for overfitting of QSAR models. Fewer molecular descriptors are generally preferred, so a punishment factor is often used in the fitness evaluation expression of PSO [15]. When the number of the candidate molecular descriptors is very large, it is inefficient to use traditional PSO algorithm directly for feature selection. The probability that only several descriptors are selected at each iteration may be very small because the number of descriptors selected in the evolution process obeys normal distribution. In order to improve the computing efficiency, the conversion of values from continuous space to discrete space is adjusted by mapping each value hitting in the interval [0, 0.05) to 1, and other values to 0. Thus, each descriptor has a probability of 1/20 of being selected and only about 1/20 of all descriptors are selected in each iteration. The probability of only several descriptors being selected will be increased dramatically.

### 2.5. Support Vector Machine (SVM)

SVM is an emerging and powerful machine learning algorithm proposed by Vapnik and co-workers in 1995 [23]. It has been extensively applied to various classification problems due to its high accuracy and its lesser proneness to overfitting than other machine learning methods. Instead of traditional empirical risk minimization, SVM achieves structural risk minimization, which results in the good generalization and avoids being trapped in local optima.

The basic theory of SVM has been presented in many references. Here only a brief description is given. A set of training points (compounds) are denoted as (***x_i_*, *y*_*i*_**), 1≤ *i* ≤ N, where *N* is the number of the training points; ***x_i_*** is the vector corresponding to data point *i* represented by a set of molecular descriptors in D-dimension space; ***y_i_*** is the class label taking value either +1 or −1. If the two classes are linearly separable, there exists a hyperplane that can separate the set by leaving all the vectors of the same class on the same side. The ultimate aim of SVM classification is to find an optimal separating hyperplane (OSH) as the decision surface to separate two classes of patterns with maximal margin. The optimal hyperplane *H* is defined mathematically by Equation (4)

(4) w·x+b=0

where ***w*** is the weight vector normal to the separating hyperplane, ***b*** is the threshold. SVM constructs two parallel hyperplanes (*H_1_* and *H_2_*) on each side of the maximal separating hyperplane that maximizes the distance between the two parallel hyperplanes. The vectors situated on two hyperplanes are called support vectors, which are used to define the separating hyperplane. Any points that fall on or above H_1_ belong to class +1, and any data points that fall on or below H_2_ belong to class −1, which can be represented as follows:

(5) $$\text{w}\cdot {\text{x}}_{\text{i}}+\text{b}\geq +1\;\text{for}\;y_{i}=+1;\;\text{class}\;1\;(\text{sensitizer})$$

(6) $$\text{w}\cdot {\text{x}}_{\text{i}}+\text{b}\leq -1\;\text{for}\;y_{i}=-1;\;\text{class}\;2\;(\text{non-sensitizer})$$

The distance from the hyperplane to any point on H_1_ is 1/||***w***||, where ||***w***|| is the Euclidean norm of ***w***. The margin of the separating hyper-plane is calculated as 2/||***w***||. The OSH has the largest margin among separating hyper-planes with the constrained optimization $\underset{\text{w}}{\text{min}}||\text{w}|{|}^{2}$ subject to inequalities (5) and (6). After the determination of ***w*** and ***b***, the classification can be realized by Equation (7):

(7) sign(w·x+b)

In most cases, the data are not linearly separable, where no linear OSH exists in the current dimensional space. Therefore, the data are nonlinearly mapped into a high-dimensional feature space where linear separation can be performed. The transform can be done by using a kernel function *K* (***x_i_***, ***x_j_***) = *Φ*(***x_i_***)·*Φ*(***x_j_***). Gaussian radial basis function, *K* (***x_i_***, ***x_j_***) = ***e***^−||***x_i_***–***_j_***||^2^ / 2σ^2^^ is one of the commonly used kernel functions. Linear support vector machine is then applied to this feature space, and the decision function is given as follows:

(8) $$\text{f}(\text{x})=\text{sign}(\sum_{i=1}^{N}{\text{y}}_{i}{\text{α}}_{i}K({\text{x}}_{i},{\text{x}}_{j})+\text{b})$$

where the coefficients ***α_i_*** and ***b*** are determined by maximizing the following Lagrange expression:

(9) $${\text{L}}_{\text{D}}=\sum_{i=1}^{l}{\text{α}}_{i}-\frac{1}{2}\sum_{i=1}^{l}\sum_{j=1}^{l}{\text{α}}_{i}{\text{α}}_{j}{\text{y}}_{i}{\text{y}}_{j}\cdot K({\text{x}}_{i},{\text{x}}_{j})$$

where ***α_i_*** ≥ 0 and $\sum_{\text{i}=1}^{\text{N}}{\text{α}}_{\text{i}}{\text{y}}_{\text{i}}=0$. The above equation can be solved numerically using quadratic programming techniques under Karush-Kuhn-Tucker(KKT) conditions to obtain the Lagrange multipliers ***α_i_***, together with ***w*** and ***b***.

Two parameters *C* and *σ* are very important to the performance of SVM. Parameter *C* represents the penalty cost, which influences the classification outcome. Parameter *σ* affects the partitioning outcome in the feature space. Ten-fold cross validation procedure was implemented to obtain the appropriate *C* and *σ*.

### 2.6. Implementation

The PSO algorithm and related programs were implemented in the Java programming language, running on the Java (TM) 2 Runtime Environment, Standard Edition (build 1.5.0_02-b09). The Java package of libsvm (version 2.8) [24] used in this work is freely available online.

### 2.7. Assessment of Results

In order to evaluate the prediction performance of SVM models, we define and compute the classification accuracy, sensitivity and specificity by the methods reported in Ref. [25]. The formulations are as follows:

(10) $$\text{Accuracy}=\frac{\text{TP}+\text{TN}}{\text{TP}+\text{FN}+\text{TN}+\text{FP}}\times 100\%$$

(11) $$\text{Sensitivity}=\frac{\text{TP}}{\text{TP}+\text{FN}}\times 100\%$$

(12) $$\text{Specificity}=\frac{\text{TN}}{\text{TN}+\text{FP}}\times 100\%$$

In Equations (10)–(12), TP is the number of true positives (sensitizers); FN, the number of false negatives (non-sensitizers); TN, the number of true negatives; and FP, the number of false positives.

## 3. Results and Discussion

### 3.1. LLNA data set

As seen in Table 1, the LLNA data set was randomly divided into a training set with 76 sensitizers and 32 non-sensitizers and a test set with 43 sensitizers and 11 non-sensitizers. Based on the training set, 123 molecular descriptors remained after preprocessing according to Section 2.3. Then, SVM combined with PSO algorithm was implemented. The PSO was set with 30 particles and 100 iterations. In each evaluation, a descriptor got a hit if this descriptor was selected. The descriptor selected more often will get more hits. Ten-fold cross validation was carried out against the training set, and the highest cross validation accuracy was used to determine the most appropriate set of molecular descriptors. In the end, six out of 123 molecular descriptors (listed in Table 2) were selected. The two most important parameters of SVM were also determined, i.e., *C*=15.81 and *σ* =14.13. The highest classification accuracy of cross validation against the training set is 83.33%. The classification accuracies, sensitivities and specificities of the training set and test set were all shown in Table 3.

Although skin sensitization is a complex toxicological phenomenon and its biomolecular processes have not been fully understood, previous studies have indicated the ability of active agents to cause immune response is related to skin permeability and the production of immunological conjugates with endogenous macromolecules [5,26]. Chemical reactivity, molecular size and skin permeability are important determinants for skin sensitization [27]. Known from Table 2, nCL represents the number of chlorine atoms in the molecule. The specific atom or group may be related to the combination or reaction of chemicals with skin protein. MAXDN and MATS1e characterize the molecular electronic structure, which may influence the electrostatic interactions between the chemical and protein. The binding of a chemical with skin proteins is considered as the rate-determing step for skin sensitization induction, where the chemical behaves as an electrophile and the protein as a nucleophile [28]. MATS2m and BELm1 are descriptors concerning molecular size, which may influence the skin penetration of compounds. The active agents causing skin sensitization are relatively small molecules. MLOGP, Moriguchi octanol-water partition coefficient, is an indicator of hydrophobic properties, which has been correlated to transport properties of a molecule, long-range ligand-receptor recognition and subsequent binding [26].

Fedorowicz [5] has also investigated the original LLNA data set (including 132 sensitizers and 46 non-sensitizers) with logistic regression and the DEREK expert system. The classification results for the training set with logistic regression and prediction results for the whole data set with DEREK reported in Ref. [5] are shown in Tables 4 and 5, respectively. For rationality, the reported results with logistic regression were compared to the classification results of this paper for the training set, while the reported results with DEREK were compared to the prediction results of this paper for the test set. Seen from Tables 4 and 5, the SVM classifier combined with PSO algorithm in this paper improved the results greatly, especially for the classification specificity. It has been explained in Ref. [5] that the very low specificity was resulted from the substantially unbalanced size of samples, i.e., the ratio of sensitizers largely overwhelming that of the non-sensitizers. However, the specificity in this paper attained 87.50% (50.00% with logistic regression in Ref. [5]) and 72.73% (32.60% with DEREK in Ref. [5]) for the training set and test set, respectively. The experimental and estimated skin sensitivities are listed in Table 6.

### 3.2. GPMT Data Set

For the GPMT data set, the same procedures as for the LLNA data set were carried out. After preprocessing according to Section 2.3, 127 molecular descriptors remained. Then, the SVM algorithm combined with PSO was implemented, and five molecular descriptors (listed in Table 7) were selected from the remaining 127 molecular descriptors. Two SVM parameters, i.e., *C*=45.63 and *σ* =1.90 were determined by 10-fold cross validation based on the training set. The total accuracy of the 10-fold cross validation is 90.16%. For the training set, the sensitizers were all classified correctly, and five non-sensitizers were mistaken as sensitizers. According to Equations (10) – (12), the classification accuracy, sensitivity and specificity are 91.80%, 100.00% and 64.29%, respectively. For the test set, only one sensitizer and two non-sensitizers were wrongly assigned. Table 8 lists the statistical parameters.

From Tables 3 and 8, we can see that the wrong classification ratio of non-sensitizers is larger than that of sensitizers for both LLNA and GPMT data sets. The unbalanced ratio (nearly 1:3) of non-sensitizers to sensitizers may be responsible for the worse classification performances for non-sensitizers than those for sensitizers. It is indicated that the non-sensitizers may be prone to be falsely predicted as sensitizers. On the contrary, if the dataset contains many more non-sensitizers than sensitizers, the QSPR model will also give biased classification results. For example, Roberts *et al.* [28] have validated the TIMES-SS (TImes MEtabolism Simulator) expert system platform used for predicting skin sensitization with 40 chemicals, consisting of 24 non-sensitizers and 16 sensitizers. TIMES-SS was able to predict non-sensitizers reasonably well (the prediction accuracy of 87.5%), while it predicted sensitizers very pooly (the prediction accuracy of 56.0%).

From Table 7, the selected five molecular descriptors come from four blocks of descriptors. nDB is a constitutional descriptor indicating the number of double bonds. The π-bond electrons in double bonds are more active than the σ-bond electrons in single bonds, therefore, the molecule with more double bonds will have larger electronic cloud deformability and may be prone to combine with the target. Five kinds of chemical reaction mechanistic domain [29,30] have been proposed, including Michael acceptors, S_N_2 electrophiles, S_N_Ar electrophiles, Schiff base electrophiles and acyl transfer electrophiles. According to Roberts [31], some compounds containing an electron-deficient double bond can be confidently assigned as Michael acceptors or pro-Michael acceptors. EEig07d and EEig14d are both descriptors related to molecular dipole moments, which indicate the molecular polarity and are closely related to the hydrophobic properties and skin permeability of molecules. O-057, the number of phenol/enol/carboxyl OHs, may be responsible for the molecular polarity and hydrogen bond, which has relationship with the combination or reaction of compounds with specific group of the receptor. As described in Ref. [31], some aromatic compounds with two *meta* hydroxyl groups may follow two possible mechanisms: reaction with molecular oxygen to introduce a hydroxyl group either *ortho* or *para* to the original hydroxyl groups, and directly binding to protein via attack of a protein-centered radical. Infective-80 is a descriptor derived from biological experiment, which may reflect directly the biochemical effect of skin sensitization induction.

Fedorowicz [5] has also investigated the original GPMT data set (including 82 sensitizers and 23 non-sensitizers) with logistic regression and two expert systems TOPKAT and DEREK. The reported classification results for the training set with logistic regression in Ref. [5] are listed in Table 9, and the reported prediction results for the whole GPMT data set with TOPKAT and DEREK are shown in Table 10. For comparison, the reported results with logistic regression were compared to the classification results of this paper for the training set, while the reported results with TOPKAT and DEREK were compared to the prediction results of this paper for the test set. As seen from Tables 9 and 10, the prediction performances of the method in this paper are far superior to those in the literature, especially for the specificity. However, expert systems such as DEREK and TIMES-SS have been recently improved by modifing the alerts describing the skin sensitization potential or considering more mechanisitic knowledge and new rules for chemicals [28,32]. Therefore, better results than those of previously reported in the literature may be achieved if the prediction is conducted with the improved expert systems. Golla *et al.* [27] built neural network models using 25, 25 and 22 molecular descriptors for LLNA, GPMT and BgVV data sets with 358, 307 and 251 compounds, respectively. The classification accuracies for the above mentioned three data sets are 90%, 95% and 90%, respectively. Compared with Ref. [27], this paper obtained the classification accuracy (for the training set) of 95.37% for LLNA data set and 91.80% for GPMT data set using only five or six molecular descriptors. The estimated skin sensitivities were listed in Table 6.

Seen from Table 6, there are ten inconsistent values in the experimental results of 92 compounds with both LLNA and GPMT data. The accuracy (or concordance) of experimental results obtained from two different test procedures for assessing skin sensitization is 89.13%. In 1999, the Interagency Coordinating Committee on the Validation of Alternative Methods (ICCVAM), with support from the National Toxicology Program Interagency Center for the Evaluation of Alternative Toxicological Methods (NICEATM), validated the experimental procedures by comparing LLNA data for 97 chemicals to the available GPMT data, and also obtained an accuracy of 89% [27]. From the above analysis, we may roughly assume that the experimental accuracy of skin sensitization is close to 89%. The prediction accuracies (for the test set) of LLNA and GPMT data sets using PSO optimized SVM in this paper were 88.89% and 90.32% respectively, which are in good agreement with the experimental accuracy.

## 4. Conclusions

This paper has investigated the skin sensitization against LLNA and GPMT data sets by particle swarm optimized support vector machine. The classification accuracies, sensitivities and specificities for both data sets were all satisfactory and largely improved compared to those obtained by logistic regression and the expert systems reported in the literature. This study has confirmed that the quantitative structure-activity relationship approach can be a promising complement to animal testing in the area of hazard identification only if a reasonable QSAR model has been constructed. The SVM classifier built in this paper can be used to assess the skin sensitization for environmental chemicals.

## Figures and Tables

**Table 1.** The composition of LLNA and GPMT data sets.

	**LLNA**			**GPMT**		

	**Tr*a***	**Te*b***	**Total**	**Tr*a***	**Te*b***	**Total**
Sensitizer (+)	76	43	119	47	24	71
Non-sensitizer (−)	32	11	43	14	7	21
Total	108	54	162	61	31	92

^a^ Tr represents the training set;

^b^ Te represents the test set.

**Table 2.** Six most important molecular descriptors selected by PSO for SVM classification for LLNA data set.

**Descriptor symbol**	**Descriptor block**	**Definition**
nCL	Constitutional descriptors	Number of chlorine atoms
MAXDN	Topological descriptors	Maximal electrotopological negative variation
MATS1e	2D autocorrelations	Moran autocorrelation-lag 1/weighted by atomic Sanderson electronegativities
MATS2m	2D autocorrelations	Moran autocorrelation-lag 2/weighted by atomic masses
BELm1	Burden eigenvalues	Lowest eigenvalue n. 1 of Burden matrix/weighted by atomic masses
MLOGP	Molecular properties	Moriguchi octanol-water partition coeff. (logP)

**Table 3.** Performance of SVM classifier combined with PSO for the skin sensitization of LLNA data set.

	**TP**	**FN**	**TN**	**FP**	**Accuracy**	**Sensitivity**	**Specficity**
Training set	75	1	28	4	95.37%	98.68%	87.50%
Test set	40	3	8	3	88.89%	93.02%	72.73%

**Table 4.** Comparison the classification performances of this paper with those reported in previous studies on the training set of LLNA data set.

	**Accuracy**	**Sensitivity**	**Specificity**
Logistic regression [5]	83.20%	94.70%	50.00%
This paper	95.37%	98.68%	87.50%

**Table 5.** Comparison the prediction performances of this paper with those reported in previous studies on the test set of LLNA data set.

	**Accuracy**	**Sensitivity**	**Specificity**
DEREK[5]	73.00%	87.10%	32.60%
This paper	88.89%	93.02%	72.73%

**Table 6.** The investigated compounds and their experimental and estimated skin sensitivities.

**ID**	**Compounds**	**CAS**	**LLNA**		**GPMT*c***	

			**Exp.**	**Calc.**	**Exp.**	**Calc.**
1	Propylene glycol	57-55-6	−1	−1	−1	−1
2 *a*	Hexane	110-54-3	−1	−1	-	-
3 *b*	Lactic acid	50-21-5	−1	−1	−1	−1
4	Phenol	108-95-2	−1	−1	-	-
5 *b*	Resorcinol	108-46-3	−1	1	−1	1
6	Chlorobenzene	108-90-7	−1	−1	−1	−1
7	Ethyl methanesulfonate	62-50-0	−1	−1	-	-
8	4-Chloroaniline	106-47-8	−1	−1	1	1
9	1-Bromobutane	109-65-9	−1	−1	-	-
10	4-Aminobenzoic acid	150−13-0	−1	−1	−1	−1
11 *b*	4-Hydroxybenzoic acid	99-96-7	−1	−1	−1	−1
12 *b*	Salicylic acid	69-72-7	−1	−1	−1	−1
13 *a*	2-Hydroxypropyl methacrylate	923-26-2	−1	−1	−1	−1
14 *b*	Tartaric acid	87-69-4	−1	−1	−1	−1
15	Methyl salicylate	119-36-8	−1	−1	−1	−1
16 *a*	Geraniol	106-24-1	−1	1	−1	−1
17	6-Methylcoumarin	92-48-8	−1	−1	−1	−1
18	1-Bromohexane	111-25-1	1	1	1	1
19	Benzocaine	94-09-7	−1	−1	1	1
20 *a*	Sulfanilamide	63-74-1	−1	−1	−1	1
21	Propylparaben	94-13-3	−1	−1	−1	−1
22 *a*	di-2-Furanylethanedione	492-94-4	−1	−1	-	-
23	2,4-Dichloronitrobenzene	611-06-3	−1	−1	−1	−1
24 *a*	Dimethyl isophthalate	1459-93-4	−1	−1	−1	−1
25	1-Bromononane	693-58-3	−1	1	-	-
26	2-Nitrofluorene	607-57-8	−1	−1	-	-
27	Phthalic acid diethyl ether	84-66-2	−1	−1	-	-
28 *a**b*	5,5-Dimethyl-3-(mesyloxymethyl) dihydro-2(3H)-furanone	154750-22-8	−1	−1	−1	1
29	2-Acetamidefluorene	53-96-3	−1	−1	-	-
30	*N*’-(4-methylcyclohexyl)-*N*-(2-chloroethyl)-*N*- nitrosourea	13909-09-6	−1	−1	-	-
31	3-(Benzenesulfonyloxymethyl)-5,5-dimethyl- dihydro-2(3*H*)-furanone	154750-24-0	−1	−1	-	-
32	Benzoyloxy-3,5-benzene dicarboxylic acid	102059-70-1	−1	−1	1	1
33 *b*	5,5-Dimethyl-3-(tosyloxymethyl)-dihydro- 2(3*H*)-furanone	154060-50-1	−1	−1	−1	−1
34	5,5-Dimethyl-3-(methoxybenzenesulfonyloxy- methyl)dihydro-2(3*H*)-furanone	154750-23-9	−1	−1	1	1
35	3-(Chlorobenzenesulfonyloxymethyl)-5,5- dimethyldihydro-2(3*H*)-furanone	154750-28-4	−1	−1	-	-
36 *b*	5,5-Dimethyl-3-(nitrobenzenesulfonyloxy- methyl)dihydro-2(3*H*)-furanone	154750-29-5	−1	−1	1	1
37 *b*	Octadecylmethane sulfonate	31081-59-1	−1	−1	1	1
38 *a*	*α*-Trimethylammonium-4-tolyloxy-4-benzene- sulfonate	264869-81-0	−1	−1	1	1
39 *a*	Hydrocortisone	50-23-7	−1	−1	-	-
40	Tixocortol-21-pivalate	55560-96-8	−1	−1	-	-
41	Kanamycin	8063-07-8	−1	−1	−1	−1
42 *a**b*	Streptomycin	57-92-1	−1	−1	1	1
43 *a*	Neomycin	1405-10-3	−1	−1	−1	−1
44 *b*	Ethylenediamine	107-15-3	1	1	1	1
45 *a*	*β*-Propiolactone	57-57-8	1	1	-	-
46 *a*	Pyridine	110-86-1	1	1	-	-
47 *a*	2,3-Butanedione	431-03-8	1	1	-	-
48 *a*	Aniline	62-53-3	1	1	1	1
49	*N*, *N*-dimethyl-1,3-propanediamine	109-55-7	1	1	1	1
50	*N*-nitroso-*N*-methylurea	684-93-5	1	1	-	-
51	Diethylenetriamine	111-40-0	1	1	1	1
52	4-Vinylpyridine	100-43-6	1	1	-	-
53 *a*	*p*-Xylene	106-42-3	1	1	-	-
54	1,4-Benzoquinone	106-51-4	1	1	1	1
55 *a*	3-Phenylenediamine	108-45-2	1	1	1	1
56 *a*	4-Phenylenediamine	106-50-3	1	1	1	1
57	1-Thioglycerol	96-27-5	1	1	1	1
58 *b*	3-Aminophenol	591-27-5	1	1	1	1
59 *a*	2-Aminophenol	95-55-6	1	1	1	1
60	Hydroquinone	123-31-9	1	1	1	1
61	Methyl methanesulfonate	66-27-3	1	1	-	-
62	2-Hydroxyethyl acrylate	818-61-1	1	1	1	1
63 *a*	*N*-Ethyl-*N*-nitrosourea	759-73-9	1	1	-	-
64	3-Methylcatechol	488-17-5	1	1	-	-
65 *a**b*	4-Methylcatechol	452-86-8	1	1	1	1
66	Dimethyl sulfate	77-78-1	1	1	-	-
67 *a*	5,5-Dimethyl-3-methylenedihydro-2(3*H*)- furanone	29043-97-8	1	1	1	1
68 *a*	Butyl glycidyl ether	2426-08-6	1	1	1	1
69 *a*	Cinnamic aldehyde	104-55-2	1	1	1	1
70	2-Methoxy-4-methylphenol	93-51-6	1	1	1	1
71 *a*	Benzoyl chloride	98-88-4	1	1	1	1
72	1-Methyl-3-nitro-1-nitrosoguanidine	70-25-7	1	1	-	-
73 *a*	Phthalic anhydride	85-44-9	1	1	1	1
74	3,4-Dihydrocoumarin	119-84-6	1	1	-	-
75	4-Allylanisole	140-67-0	1	1	1	1
76 *a**b*	5-Chloro-2-methyl-4-isothiazolin-3-one	26172-55-4	1	1	1	1
77 *a*	4-Nitroso-*N*,*N*-dimethylaniline	138-89-6	1	1	1	1
78	1,2-Benzisothiazol-3(2*H*)-one	2634-33-5	1	1	1	1
79 *b*	Citral	5392-40-5	1	1	1	1
80	Diethyl sulfate	64-67-5	1	1	-	-
81 *a*	2-Methyl-4,5-trimethylene-4-isothiazolin-3-one	82633-79-2	1	1	1	1
82 *a*	1-Ethyl-3-nitro-1-nitrosoguanidine	4245-77-6	1	1	-	-
83	1-Chlorononane	2473-01-0	1	1	-	-
84	Eugenol	97-53-0	1	1	1	1
85	Isoeugenol	97-54-1	1	1	1	1
86 *b*	Dihydroeugenol	2785-87-7	1	1	1	1
87 *b*	2-Mercaptobenzothiazole	149-30-4	1	1	1	1
88	Benzyl bromide	100-39-0	1	1	-	-
89	4-Nitrobenzyl chloride	100-14-1	1	1	1	1
90 *a*	Hydroxycitronellal	107-75-5	1	1	1	1
91	1-Propyl-3-nitro-1-nitrosoguanidine	13010-07-6	1	1	-	-
92 *a*	Nonanoyl chloride	764-85-2	1	1	-	-
93	3,5,5-Trimethylhexanoyl chloride	36727-29-4	1	1	1	1
94	3-Methyleugenol	186743-26-0	1	1	-	-
95 *a*	5-Methyleugenol	186743-25-9	1	1	-	-
96	6-Methyleugenol	186743-24-8	1	1	-	-
97	2,4,6-Trichloro-1,3,5-triazine	108-77-0	1	1	-	-
98 *a*	5,5-Dimethyl-3-(thiocyanatomethyl)-dihydro- 2(3*H*)-furanone	154750-32-0	1	1	1	1
99	2,4-Dinitrofluorobenzene	70-34-8	1	1	-	-
100 *a*	Bbenzene-1,3,4-tricarboxylic anhydride	552-30-7	1	1	1	1
101	2,4,5-Trichlorophenol	95-95-4	1	1	-	-
102 *a*	Ethylene glycol dimethacrylate	97-90-5	1	1	−1	−1
103 *a**b*	Phenyl benzoate	93-99-2	1	1	1	1
104 *b*	2,4-Dinitrochlorobenzene	97-00-7	1	1	1	1
105 *b*	5,5-Dimethyl-3-(bromomethyl)dihydro- 2(3*H*)-furanone	154750-20-6	1	1	1	1
106	1-Iodohexane	638-45-9	1	1	-	-
107	Propyl gallate	121-79-9	1	1	1	1
108	2-Chloromethylfluorene	91679-67-3	1	1	-	-
109 *b*	4-Nitrobenzyl bromide	100-11-8	1	1	1	1
110 *b*	Hexyl cinnamic aldehyde	101-86-0	1	1	1	1
111 *b*	Isophorone diisocyanate	4098-71-9	1	1	1	1
112	2,4-Dinitrothiocyanobenzene	1594-56-5	1	1	1	1
113 *a*	3-Methoxyphenylbenzoate	5554-24-5	1	1	-	-
114	1-Chlorotetradecane	2425-54-9	1	1	-	-
115 *a*	1-Bromoundecane	693-67-4	1	1	-	-
116	3-Acetylphenyl benzoate	139-28-6	1	1	1	1
117 *b*	Tetramethyl thiuram disulfide	137-26-8	1	1	1	1
118 *b*	Benzoyl peroxide	94-36-0	1	1	1	1
119 *a*	Picryl chloride	88-88-0	1	1	1	1
120 *a**b*	1-Bromododecane	143-15-7	1	1	1	1
121	Methylene diphenyl diisocyanate	101-68-8	1	1	1	1
122 *a*	1-Chloromethylpyrene	1086-00-6	1	1	-	-
123 *a*	Benzopyrene	50-32-8	1	1	-	-
124	1-Iodononane	4282-42-2	1	1	-	-
125	7,12-Dimethylbenzanthracene	57-97-6	1	1	-	-
126 *a*	1-Bromotridecane	765-09-3	1	1	-	-
127 *a*	Dodecyl methanesulfonate	51323-71-8	1	1	1	1
128	Methyl dodecanesulfonate	2374-65-4	1	1	1	1
129 *a*	12-Bromo-1-dodecanol	3344-77-2	1	1	-	-
130	1,2-Dibromo-2,4-dicyanobutane	35691-65-7	1	1	1	1
131	Pentachlorophenol	87-86-5	1	1	-	-
132 *a*	Dodecylthiosulfonate	127089-67-2	1	1	1	1
133	3-Methylcholantrene	56-49-5	1	1	-	-
134	*α*-Naphthoflavone	604-59-1	1	1	-	-
135	*β*-Naphthoflavone	6051-87-2	1	1	-	-
136 *a*	Hexadecanoyl chloride	112-67-4	1	1	-	-
137	7-Bromotetradecane	74036-97-8	1	1	-	-
138 *a*	1-Bromotetradecane	112-71-0	1	1	-	-
139	12-Bromododecanoic acid	73367-80-3	1	1	-	-
140	2-(*N*-Acetoxy-acetamido)fluorene	6098-44-8	1	1	-	-
141	Octyl gallate	1034-01-1	1	1	-	-
142	1-Chlorooctadecane	3386-33-2	1	1	-	-
143	1-Bromopentadecane	629-72-1	1	1	-	-
144 *b*	Oxazolone	1564-29-0	1	1	1	1
145 *b*	Abietic acid	514-10-3	1	1	1	1
146 *a*	Octadecanoyl chloride	112-76-5	1	1	-	-
147 *b*	1-Bromohexadecane	112-82-3	1	1	1	1
148	2-Bromotetradecanoic acid	10520-81-7	1	1	-	-
149	Methyl hexadec-2-ene sulfonate	54612-23-6	1	1	1	1
150	Chlorpromazine	50-53-3	1	1	1	1
151 *a*	1-Bromoheptadecane	3508-00-7	1	1	-	-
152 *a*	1-Iodotetradecane	19218-94-1	1	1	-	-
153	1-Bromooctadecane	112-89-0	1	1	-	-
154	Penicillin G	61-33-6	1	1	1	1
155	Clotrimazole	23593-75-1	1	1	-	-
156	Tetrachlorosalicylanilide	1154-59-2	1	1	1	1
157	1-Iodohexadecane	544-77-4	1	1	-	-
158 *a*	1-Iodooctadecane	629-93-6	1	1	-	-
159 *b*	Imidazolidinyl urea	39236-46-9	1	1	1	1
160	Dimethyl sulfostearate	99785-70-3	1	1	-	-
161 *b*	Sulfanilic acid	121-57-3	−1	−1	1	1
162 *a*	Isononanoyloxybenzene sulfonate	109363-00-0	1	1	1	1

^a^ Compounds making up of the test set of the LLNA data set;

^b^ Compounds making up of the test set of the GPMT data set;

^c^ “-” denotes no GPMT data available.

**Table 7.** Five molecular descriptors selected by PSO for SVM classification of GPMT data set.

**Descriptor symbol**	**Descriptor block**	**Definition**
nDB	Constitutional descriptors	Number of double bonds
EEig07d	Edge adjacency indices	Eigenvalue 07 from edge adj. matrix weighted by dipole moments
EEig14d	Edge adjacency indices	Eigenvalue 14 from edge adj. matrix weighted by dipole moments
O-057	Atom-centred fragments	Phenol / enol / carboxyl OH
Infective-80	Molecular properties	Ghose-Viswanadhan-Wendoloski antiinfective-like index at 80%

**Table 8.** Performance of SVM classifier combined with PSO for the skin sensitization of GPMT data set.

	**TP**	**FN**	**TN**	**FP**	**Accuracy**	**Sensitivity**	**Specficity**
Training set	47	0	9	5	91.80%	100.00%	64.29%
Test set	23	1	5	2	90.32%	95.83%	71.43%

**Table 9.** Comparison the classification performances of this paper with those reported in previous studies on the training set of GPMT data set.

	**Accuracy**	**Sensitivity**	**Specificity**
Logistic regression	87.60%	98.80%	47.80%
This paper	91.80%	100.00%	64.29%

**Table 10.** Comparison the prediction performances of this paper with those reported in previous studies on the test set of GPMT data set.

	**Accuracy**	**Sensitivity**	**Specificity**
TOPKAT	73.30%	75.60%	65.20%
DEREK	82.90%	92.70%	47.80%
This paper	90.32%	95.83%	71.43%
